# Pyriform Sinus Fistula in the Fetus and Neonate: A Systematic Review of Published Cases

**DOI:** 10.3389/fped.2020.00502

**Published:** 2020-08-25

**Authors:** Tong Chen, Jianglong Chen, Qingfeng Sheng, Linlin Zhu, Zhibao Lv

**Affiliations:** Department of General Surgery, Shanghai Children's Hospital, Shanghai Jiao Tong University, Shanghai, China

**Keywords:** diagnosis, fetus, neonate, pyriform sinus fistula, treatment

## Abstract

**Purpose:** To provide recommendations for the diagnosis and treatment of pyriform sinus fistula (PSF) in the fetus and neonate through a systematic review of the published literature.

**Methods:** PubMed and Embase (1968–2019) were searched, and additional publications were obtained by searching the references by hand. The two reviewers assessed all papers and extracted the following variables: demographics, clinical features, diagnostic tests, interventions, and prognoses.

**Results:** Forty-two papers were included, comprising a total of 158 cases. PSF presented almost exclusively on the left side (95.56%). Patients usually presented with a neck mass (100%) and respiratory distress (43.18%). The false-negative rate (FNR) of prenatal ultrasonography (US) was significantly higher than that of prenatal magnetic resonance imaging (MRI) (*P* < 0.01). For the diagnosis of PSF in neonates, computerized tomography (CT) and MRI were the most accurate diagnostic modalities. *Ex utero* intrapartum treatment (EXIT) was performed during delivery in 6 patients (26.09%). Among 135 patients with a reported date of definitive surgery, 117 (86.67%) underwent surgery during the neonatal period. Complications after definitive surgery appeared in 5 patients (3.16%), and all of them recovered spontaneously within 3 months. Furthermore, recurrence occurred in 4 patients (2.53%).

**Conclusion:** In fetal cases with PSF suspected by US, MRI is necessary to confirm the diagnosis. During the neonatal period, patients with PSF typically present with a neck mass and respiratory distress, and CT/MRI appears to be the preferred diagnostic method. Definitive surgery is effective for treating neonatal PSF, with a low complication rate and low recurrence rate.

## Introduction

Pyriform sinus fistula (PSF), constituting <2% of branchial cleft anomalies (1), is a congenital entity due to abnormal development of the third or fourth branchial cleft (2). Anatomically, PSF begins at the pyriform fossa, penetrates the cricothyroid muscle, and terminates laterally in the thyroid gland (3, 4).

Approximately 80% of patients with PSF show onset in the neonatal or pediatric period (5). Children with PSF often present with recurrent neck abscesses and acute thyroiditis. In contrast, neck masses and respiratory problems are the main presentations in neonates with PSF, and tracheal compression may lead to apnea. Therefore, the diagnosis of PSF in the fetus and neonate has been widely acknowledged as a growing concern (6–10). In addition to the clinical presentations, the preferred imaging test between children and neonates is also quite different (11). Although the first PSF case reported in the literature was a 3-week-old neonate (12), the majority of reported PSF cases are children. Collectively, the underreported condition of PSF in the fetus and neonate has given rise to frequently delayed diagnosis or misdiagnosis. Furthermore, there are few recommendations for the diagnosis and treatment of this entity, and the preferred method of definitive surgery is yet to be determined. Therefore, we performed a review of all published fetal and neonatal cases of PSF to summarize the clinical characteristics and to investigate the preferred options for diagnosis and treatment.

## Materials and Methods

### Registration and Search Strategy

**Figure 1:**
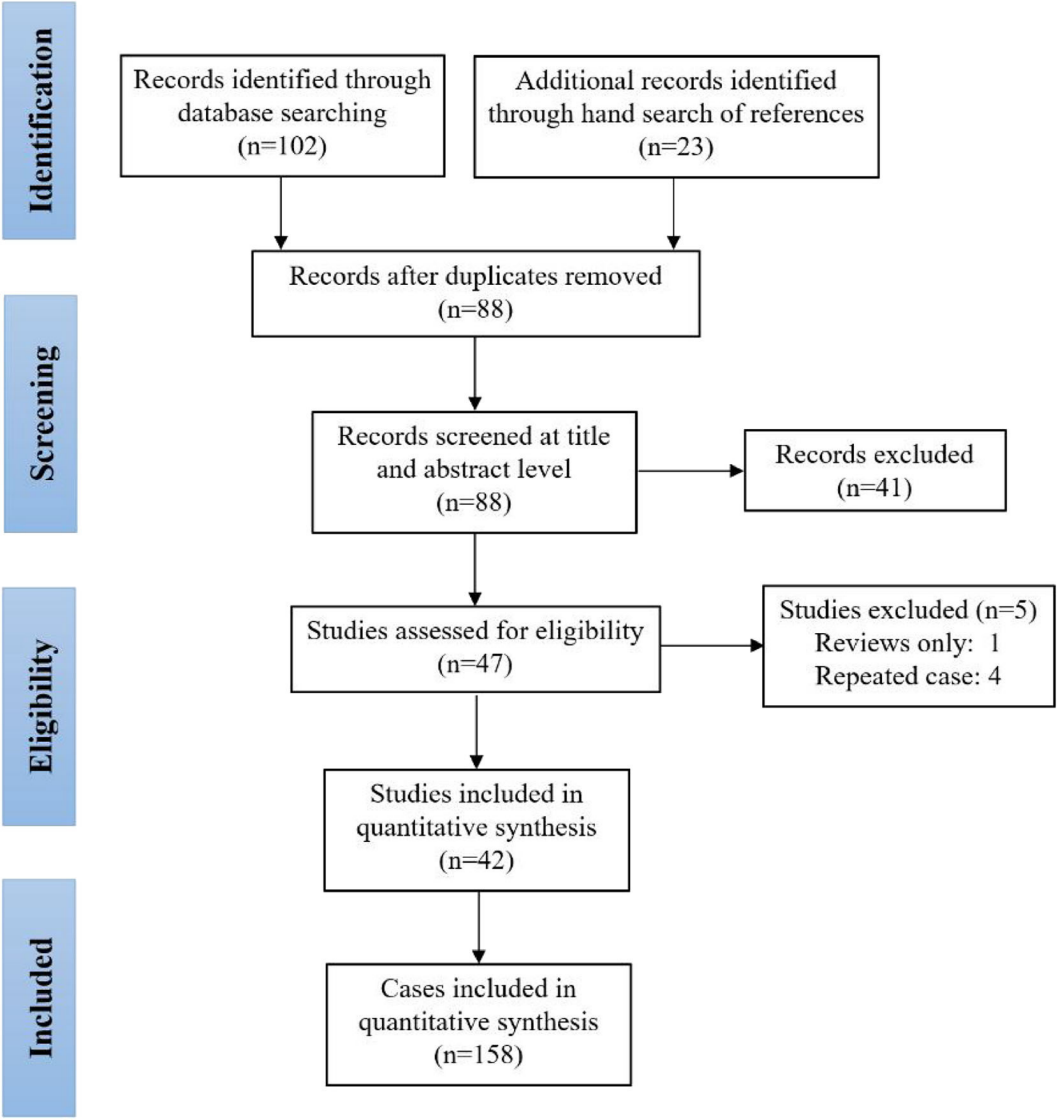
Flowchart of the search strategy and included studies.

This systematic review was registered in the International Prospective Register of Systematic Reviews (PROSPERO)—CRD42020160755. Cases were identified by detailed searches of PubMed and Embase with the following word combinations: (“piriform sinus tract” OR “piriform sinus fistula” OR “piriform sinus cyst” OR “third branchial cleft anomaly” OR “fourth branchial cleft anomaly”) AND (“prenatal” OR “fetus” OR “*in utero*” OR “neonate” OR “newborn”). Included papers were restricted to those published between 1968 and 2019. Furthermore, we did not evaluate papers without English-language abstracts. The searching and screening of papers and abstracts were conducted independently by two reviewers (TC, JC), and the references of selected papers were also checked to identify other eligible studies. Inconsistent results found by the two reviewers were further assessed and discussed to reach a consensus. Figure 1 illustrates the detailed flowchart of the search strategy, and 42 studies were included in this review (6, 9–11, 13–50).

### Inclusion Criteria

We included patients who were diagnosed with PSF by imaging or endoscopic procedures during the prenatal or neonatal period and who had surgically confirmed PSF reported in the study. Thus, patients newly diagnosed with PSF by imaging or endoscopic procedures after the neonatal period were excluded.

### Diagnostic Criteria

The diagnosis of PSF in the fetus was made when ultrasonography (US) or magnetic resonance imaging (MRI) showed a single-cystic mass in the neck, usually on the left side, located near the thyroid gland beneath the laryngeal fossa. The diagnosis of PSF in the neonate was made when at least one of the following criteria was met: (1) US, computerized tomography (CT) or MRI demonstrated a simple cystic mass in the neck, especially on the left side, usually with an air-fluid level in the cyst; (2) barium esophagography delineated the fistulous tract; or (3) the inner orifice of the fistulous tract was detected by endoscopic procedures.

### Data Extraction

The two reviewers assessed all relevant papers and extracted the following variables: demographics, clinical features, diagnostic tests, treatments, postoperative pathological examination results, complications, and cases of recurrence.

### Statistical Analysis

Statistical Package for the Social Sciences software (version 22.0, Chicago, USA) was applied for data analyses. Continuous variables were recorded as the mean and standard deviation, whereas categorical variables were described as N with percentage. The false-negative rate (FNR) was computed as the number of false-negative (FN) results/(the number of true-positive [TP] results + the number of FN results). The proportions of different groups were compared using χ^2^. A two-sided confidence level of *P* < 0.05 indicated significance.

## Results

### Demographics and Clinical Features

One hundred and fifty-eight cases were enrolled in this review. The male-to-female ratio was 1.42:1, which yielded a male predominance. PSF occurred almost exclusively on the left side. Patients with PSF usually presented with various degrees of neck masses and respiratory distress (Table 1). In addition, neck infection and fever occurred in 36.36 and 11.36% of the neonates, respectively. The maximum size of the neck mass in the neonates ranged from 2.2 to 6.0 cm (mean: 4.71 cm). The mean maternal age was 27.90 ± 5.67 years, ranging from 19 to 41 years. Eight patients (5.06%) developed neck infections during the neonatal period.

**Table 1:** Characteristics of the included neonates with PSF.

**Characteristics**	***N***	**%**
Sex (*n* = 138)		
Female	57	41.30
Male	81	58.70
Age at symptom onset (*n* = 38)		
≤ 7	15	39.47
>8, ≤ 28	23	60.53
Side (*n* = 135)		
Left	129	95.56
Right	5	3.70
Bilateral	1	0.74
Delivery mode (*n* = 51)		
Eutocia	24	47.06
Cesarean	27	52.94
Gestational weeks (*n* = 58)		
≤ 36	4	6.90
>37, ≤ 40	45	77.59
>40	9	15.52
Birth weight (*n* = 41)		
Low birth weight	3	7.32
Normal birth weight	38	92.68
Presentation (*n* = 132)		
Neck mass	132	100
Respiratory distress	57	43.18
Infection	8	6.06
Fever	15	11.36

*PSF, pyriform sinus fistula*.

### Diagnostic Test

The FN result and FNR of each diagnostic test performed prenatally or postnatally are listed in Table 2. The earliest detection of a neck mass using prenatal US was during the 18th week of gestation. Overall, twenty-three patients (14.56%) were prenatally diagnosed with PSF using US and/or MRI. Prenatal US yielded a significantly higher FNR than prenatal MRI (*P* < 0.01). Furthermore, the FNR of CT or MRI performed after birth was significantly lower than that of US, barium esophagography, laryngoscopy and esophagoscopy (*P* < 0.05).

**Table 2:** Results of different diagnostic tests of the included cases.

**Diagnostic test**	**Total cases**	**FN result**	**FNR (%)**
Prenatal imaging test			
US	54	39	72.22
MRI	13	3	23.08
Neonatal test			
US	19	9	47.37
CT	98	25	25.51
MRI	27	6	22.22
Barium esophagography	93	48	51.61
Laryngoscopy or esophagoscopy	33	11	33.33

*CT, computed tomography; FN, false-negative, FNR, false-negative rate; MRI, magnetic resonance imaging; PSF, pyriform sinus fistula; US, ultrasonography*.

### Treatments and Outcomes

*Ex utero* intrapartum treatment (EXIT) was performed during delivery in 6 patients (26.09%). Preoperative incubation was conducted in 14 patients (8.86%). Twenty-five patients (15.82%) underwent incision and drainage. Among 135 patients with a reported date of definitive surgery, 117 patients (86.67%) underwent definitive surgery during the neonatal period, whereas 18 patients (13.33%) underwent definitive surgery after the neonatal period. The specific type of definitive surgery was reported in 126 cases. Endoscopic-assisted surgery was performed in 60 cases (47.62%), and traditional open neck surgery was performed in 52 cases (41.27%). In addition, fourteen patients (11.11%) underwent cauterization of the inner orifice. The results of histopathologic examinations were described in 100 cases (63.29%); among them, squamous epithelium, respiratory epithelium, and inflammatory cells constituted the majority of conditions. Additionally, thyroid, thymic, and parathyroid tissues were sometimes demonstrated. The follow-up duration ranged from 6 to 180 months (mean: 47.52 months). Complications appeared in 5 patients (Table 3), and all of them recovered spontaneously within 3 months after surgery. Furthermore, recurrence occurred in 4 patients (Table 4). Specifically, recurrence appeared in 2 patients after open neck surgery, and the other 2 patients experienced recurrence after cauterization.

**Table 3:** Complications of definitive surgeries for neonatal PSF in 5 cases.

**References**	**Sex**	**Side**	**Age at symptom onset**	**Age at the time of surgery**	**Presentation**	**Category of definitive surgery**	**Complication**	**Follow-up**
Narcy et al. (16)	Female	Left	18 days	Not reported	Neck mass, Respiratory distress	Open surgery	Left vocal paralysis	Not reported
Franciosi et al. (27)	Male	Left	4 days	Not reported	Neck mass, Respiratory distress	Open surgery	Bilateral vocal cord paralysis	Spontaneously recovered after 2 weeks
de Buys Roessingh et al. (34)	Male	Left	Birth	Not reported	Neck mass, Respiratory distress	Laryngoscopy assisted cauterization	Slight respiratory distress	Recovered within 2 weeks
Amano et al. (39)	Female	Left	Birth	7 days	Neck mass	Open surgery	Transient paralysis of the left recurrent laryngeal nerve	Spontaneously recovered
Zhu et al. (10)	Not reported	Left	Not reported	Neonatal period	Neck mass	Open surgery	Voice hoarseness	Recovered after 3 months

**Table 4:** Reported recurrences after definitive surgeries for neonatal PSF in 4 cases.

**References**	**Sex**	**Side**	**Age at symptom onset**	**Age at the time of surgery**	**Presentation**	**First definitive surgery**	**Recurrence**	**Final surgery**	**Age at the time of final surgery**
Narcy et al. (16)	Female	Left	22 days	2 months	Neck mass, respiratory distress, fever	Open surgery	Cervical abscesses (twice)	Open surgery	6 years
Joshi et al. (36)	Female	Left	34 weeks of gestation	Not reported	Neck mass	Cauterization	Mass not resolved	Open surgery	36 days
Leboulanger et al. (38)	Not reported	Not reported	Not reported	Not reported	Neck mass	CO_2_ laser cauterization	Neck mass	Open surgery	3 days after the first surgery
Hwang et al. (45)	Not reported	Not reported	Not reported	Not reported	Neck mass	Open surgery	Neck inflammation	Open surgery	Not reported

## Discussion

PSF is characterized by incomplete obliteration of the third or fourth pharyngeal pouch linked with the pyriform sinus, which ought to involute during the 7th week of gestation (12). Although severe neck infection was reported to be fatal in an adult with PSF (51), PSF usually gives rise to non-lethal outcomes in children, which reveals the benign nature of the disease. However, respiratory distress, one common presentation in neonates with PSF, has the potential to cause life-threatening complications. Delayed diagnosis or misdiagnosis of neonatal PSF is quite common due to its rarity. Therefore, it is essential to provide recommendations regarding diagnosis and treatment of neonatal PSF.

In this review, 95.56% of PSF cases presented on the left side. This left-sided predominance could be attributable to the degeneration of the right ultimobranchial body, the more complicated fistulous tract on the left side (52), and asymmetric vascular agenesis (3). As PSF is located close to the respiratory tract and may lead to high pressure in the trachea, EXIT is sometimes necessary during delivery (53). The main presentations of neonates with PSF are neck mass and respiratory distress. In contrast, PSF often presents as recurrent cervical inflammation and acute suppurative thyroiditis in children (39). One hypothesis is that the sinus tract alters the position along with aging. Specifically, the tract is situated near the pyriform sinus in the neonate, and moves lower in the neck after the neonatal period. The other hypothesis is that the cartilage of neonatal the trachea is weak and less robust during the neonatal period. The adverse impact of neonatal PSF is dependent on its size and location and on the presence of neck infections (34). After oral feeding, the size of the neck mass gradually increases in some cases (32, 44), which may lead to tracheal compression.

The accurate diagnosis of PSF in the fetus is important, because it can help clinicians preplan the timing of postnatal treatment. However, the diagnosis of fetal PSF remains a great challenge for radiologists due to the small cyst size. This review revealed 23 cases with a prenatal diagnosis of PSF (6, 7, 9, 24, 32, 54). PSF-related neck abnormality is often identified in the second or third trimester. To date, the earliest detection of PSF-related abnormality was achieved during the 18th week of gestation using prenatal US (4). Our data demonstrated that the FNR of fetal MRI was significantly lower than that of fetal US. For the differential diagnosis of PSF, lymphangioma, thyroid cysts, cystic hygroma, and esophageal cysts should be taken into consideration. Specifically, lymphangioma is often combined with other malformations, which together lead to fetal hydrops. In addition, lymphangioma is usually detected in early gestation and in the posterior cervical space (55). Thyroid cyst is characterized by round cyst in the parenchyma of the thyroid, and the wall of thyroid cyst is thinner than that of PSF. With the help of US and MRI, the accurate diagnosis of fetal PSF is possible (8, 56).

Multiple diagnostic methods, including laryngoscopy, barium esophagography, US, CT, and MRI, have been adopted for the diagnosis of neonatal PSF (52, 57–61). US is often regarded as the initial screening test. In this review, the FNR of barium esophagography was 51.61%, which was the most insensitive test for diagnosing neonatal PSF. The narrow orifice of the fistulous tract might prevent barium from penetrating, contributing to the FN results (62). Thus, it is inadvisable to perform barium esophagography for the evaluation of neonatal PSF. Both CT and MRI are considered reliable diagnostic methods to evaluate neonatal PSF (11). Compared with MRI, CT is much cheaper and more readily and widely available but yields additional radiation exposure. The diagnosis of PSF can be made when CT shows an air-fluid level in the cyst. In addition, the anatomical association between the tract and the thyroid can also be assessed using CT (63). On the other hand, MRI may distinguish the inflammatory condition of the tract but cannot detect air in the cyst (64).

**Figure 2:**
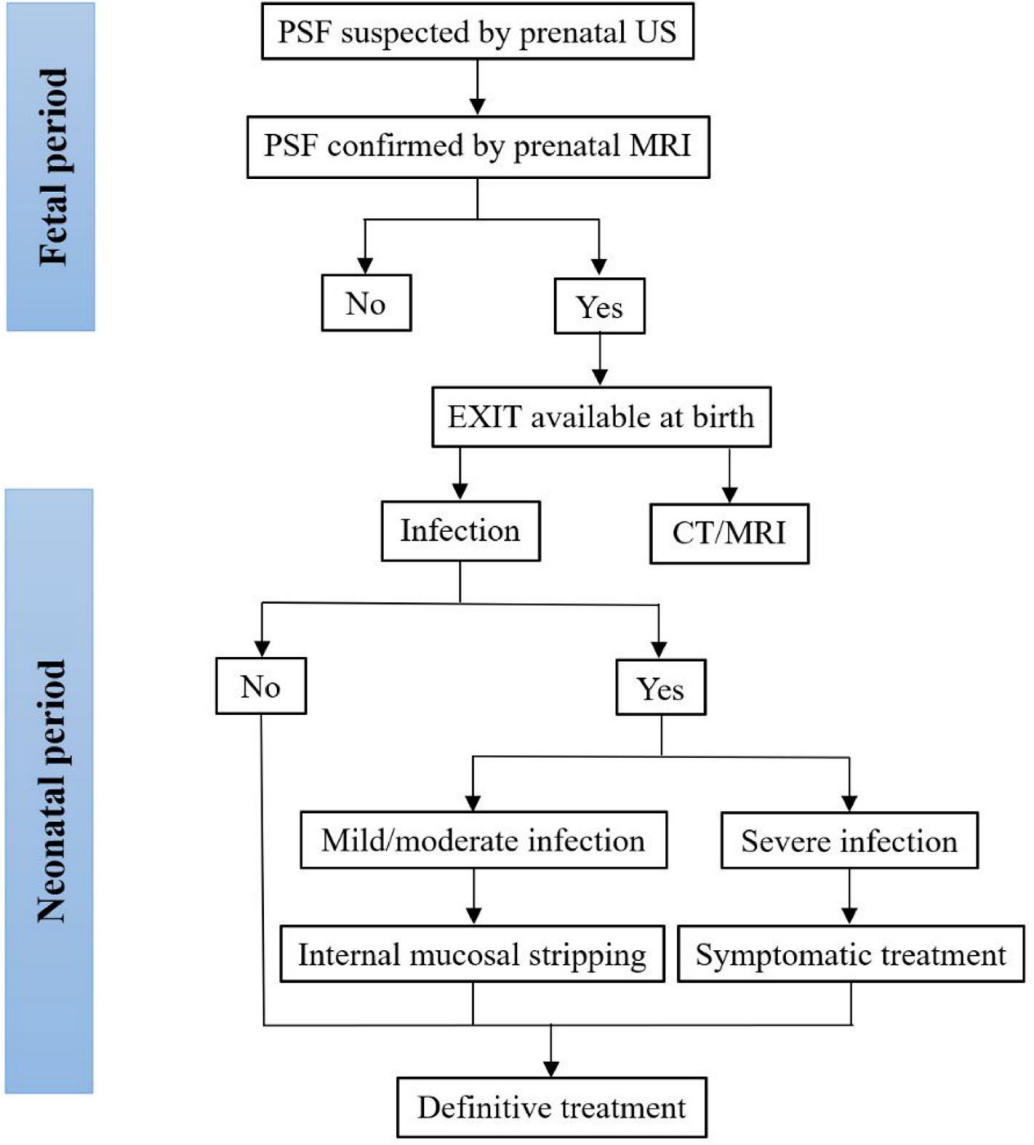
Tentative algorithm for the diagnosis and treatment of PSF in the fetus and neonate. CT, computed tomography; MRI, magnetic resonance imaging; PSF, pyriform sinus fistula; US, ultrasonography.

Treatment options for neonatal PSF depend on whether there are neck infections. Recently, it was reported that neck infections were uncommon in neonates with PSF, particularly during the period from 0 to 7 days after birth (10, 11), and the optimal timing for definitive surgery was 0–7 days after birth (10), yielding no complications or cases of recurrence (8, 46). Similarly, the majority of neonates with PSF in this review did not develop neck infections. Various definitive surgeries were demonstrated in the included papers. Traditional open neck surgery and endoscopic-assisted surgery were the two mainstay treatments for neonatal PSF. A minority of patients underwent cauterization using CO_2_ or a thulium laser. Cauterization of the internal orifice offered no skin incision requirement, easier identification of the tract and a shorter hospitalization time, and it has become popular for the treatment of PSF in children. However, it is difficult to perform endoscopic procedures in neonates, such as the placement of an endoscope inside the narrow oropharyngeal space. The tentative algorithm for the diagnosis and treatment of PSF in the fetus and neonate is plotted in Figure 2. Before the first episode of inflammation, one-stage definitive surgery can be performed. In neonates with mild or moderate infections, since inflammatory adhesions surround the tract, internal mucosal stripping should be performed to avoid postoperative complications. Fluctuations in neck swelling and skin redness suggest severe neck infection. Under this condition, incision, and drainage with broad-spectrum antibiotics should be adopted. Inflammation subsides after at least 4 weeks. Then, definitive surgery can be performed (61, 62, 65).

The current study has some limitations. First, the included cases were extracted from a large and heterogeneous collection of case reports and small case series, varying in terms of methodology and description of clinical features. Therefore, detailed statistical analyses conducted in a meta-analysis of studies are not suitable for this review. In addition, the review may be limited by potential publication bias. Finally, our recommendations were based on a small population size and should be verified by further studies with larger sample sizes, in particular, randomized control trials.

In conclusion, in fetal cases of PSF suspected by US, it is advisable to perform MRI to further confirm the diagnosis. During the neonatal period, PSF patients typically present with neck mass and respiratory distress. Both CT and MRI appear to be the preferred modalities for the diagnosis of neonatal PSF. Definitive surgery is effective for treating neonatal PSF, with a low complication rate and low recurrence rate.

## Data Availability Statement

All datasets generated for this study are included in the article/supplementary material.

## Author Contributions

TC and ZL: study design. TC, JC, and QS: collection and analysis of data. TC, JC, and LZ: manuscript writing. All authors: contributed to the article and approved the submitted version.

## Conflict of Interest

The authors declare that the research was conducted in the absence of any commercial or financial relationships that could be construed as a potential conflict of interest.
